# Repetitive DNA is associated with centromeric domains in *Trypanosoma brucei *but not *Trypanosoma cruzi*

**DOI:** 10.1186/gb-2007-8-3-r37

**Published:** 2007-03-12

**Authors:** Samson O Obado, Christopher Bot, Daniel Nilsson, Bjorn Andersson, John M Kelly

**Affiliations:** 1 Department of Infectious and Tropical Diseases, London School of Hygiene and Tropical Medicine, Keppel Street, London WC1E 7HT, UK; 2 Center for Genomics and Bioinformatics, Karolinska Institutet, Berzelius vag, S-171 77 Stockholm, Sweden

## Abstract

**Background:**

Trypanosomes are parasitic protozoa that diverged early from the main eukaryotic lineage. Their genomes display several unusual characteristics and, despite completion of the trypanosome genome projects, the location of centromeric DNA has not been identified.

**Results:**

We report evidence on the location and nature of centromeric DNA in *Trypanosoma cruzi *and *Trypanosoma brucei*. In *T. cruzi*, we used telomere-associated chromosome fragmentation and found that GC-rich transcriptional 'strand-switch' domains composed predominantly of degenerate retrotranposons are a shared feature of regions that confer mitotic stability. Consistent with this, etoposide-mediated topoisomerase-II cleavage, a biochemical marker for active centromeres, is concentrated at these domains. In the 'megabase-sized' chromosomes of *T. brucei*, topoisomerase-II activity is also focused at single loci that encompass regions between directional gene clusters that contain transposable elements. Unlike *T. cruzi*, however, these loci also contain arrays of AT-rich repeats stretching over several kilobases. The sites of topoisomerase-II activity on *T. brucei *chromosome 1 and *T. cruzi *chromosome 3 are syntenic, suggesting that centromere location has been conserved for more than 200 million years. The *T. brucei *intermediate and minichromosomes, which lack housekeeping genes, do not exhibit site-specific accumulation of topoisomerase-II, suggesting that segregation of these atypical chromosomes might involve a centromere-independent mechanism.

**Conclusion:**

The localization of centromeric DNA in trypanosomes fills a major gap in our understanding of genome organization in these important human pathogens. These data are a significant step towards identifying and functionally characterizing other determinants of centromere function and provide a framework for dissecting the mechanisms of chromosome segregation.

## Abstract 2

Centromeres in *Trypanosoma cruzi *and *Trypanosoma brucei *can be localised to regions between directional gene clusters that contain degenerate retroelements, and in the case of *T. brucei*, repetitive DNA.

## Background

Centromeres are the chromosomal loci where kinetochores are assembled. The centromere/kinetochore complex is the anchor for attachment of the microtubule spindles that facilitate segregation. Two main classes of centromere have been identified. In most eukaryotes, centromeres are 'regional' and can encompass large regions of chromosomal DNA, ranging from 0.3-15 Mb in species as diverse as plants, insects and mammals [1]. In microorganisms, regional centromeres are also extensive; in *Schizosaccharomyces pombe *they cover 35-110 kb [2]. Less common are 'point' centromeres, such as those in *Saccharomyces cerevisiae*, where specific 125 base-pair (bp) elements are sufficient for spindle attachment [3]. A few organisms, including *Caenorhabditis elegans*, lack specific centromeric domains and have holocentric chromosomes, where microtubules bind along the entire length of the chromosome [4].

Regional centromeres generally contain long stretches of repetitive DNA, often interrupted by retrotransposons. For example, the human × chromosome has a conserved core of α-satellite repeats (approximately 170 bp) stretching over 2-4 Mb and flanked by long regions with multiple retrotransposon insertions [5]. In *S. pombe*, centromeres are structured as chromosome-specific core elements, flanked by inverted repeats of approximately 3-7 kb, which in turn are flanked by more extensive outer repeats [2]. Some features of centromere organization are widespread, although there is little conservation at the level of DNA sequence [6]. The observation that inheritable neocentromeres in human cells can form at loci lacking α-satellite repeats suggests that epigenetic factors must be major determinants of centromere identity [7]. Neocentromeres have also been observed in other species, including insects and plants.

Topoisomerase-II (Topo-II) is thought to have an important role in centromere function [8-10]. During metaphase the enzyme accumulates specifically at active centromeres, where it has been implicated in maintaining kinetochore/centromere structure and decatenation of sister chromatids [9,11-13]. Decatenation involves double-stranded DNA cleavage, passage of the unbroken helix of the duplex through the gap, and re-ligation to repair the lesion [14]. This activity can be blocked by etoposide, which inhibits the re-ligation step, thereby promoting double-stranded DNA breaks in the chromosome at sites specified by Topo-II binding. As a result, etoposide has been used to map active centromeres and as a tool to explore the key role of Topo-II in centromere function [15-18]. In *Plasmodium falciparum*, Topo-II activity concentrates at single chromosomal loci that encompass 2 kb AT-rich domains previously identified as candidate centromeres [19].

Protozoan parasites of the family Trypanosomatidae are the causative agents of African sleeping sickness (*Trypanosoma brucei*), American trypanosomiasis (*Trypanosoma cruzi*) and leishmaniasis (*Leishmania *spp.), diseases that affect more than 30 million people, mainly in the developing world. Trypanosomatids are early diverging eukaryotes and share several unusual genetic traits [20]. Protein coding genes lack RNA polymerase II-mediated promoters, transcription is polycistronic and all mRNAs are post-transcriptionally modified by addition of a 5'-spliced leader RNA. Directional gene clusters often stretch over hundreds of kilobases. Trypanosomes exhibit significant intra-strain variation in chromosome size, and although generally diploid, chromosome homologues can differ considerably in length. *T. cruzi *has a haploid genome size of 55 Mb and approximately 30 chromosomes. The precise number has been difficult to determine because of size heterogeneity, recombination and, in some instances, triploidy [21,22]. In *T. brucei *(haploid genome size 25 Mb), unusually there are 3 chromosome classes; 11 homologous pairs (1-6 Mb) that contain the actively expressed genes, 3-5 intermediate-sized chromosomes (0.2-0.7 Mb) that contain some variable surface glycoprotein (*VSG*) expression sites, but lack housekeeping genes, and approximately 100 minichromosomes (approximately 0.1 Mb) which may be a reservoir for *VSG *sequences. The trypanosome sequencing projects have been completed [21,23]. A striking feature of genome organization is the high level of synteny. However, sequence elements that could have a role in chromosome segregation were not recognized. Furthermore, there are no obvious homologues of the core proteins that display constitutive centromere location in other eukaryotes [1,23].

To identify *T. cruzi *sequence elements with centromeric properties, we previously used telomere-associated chromosome fragmentation to delineate a region of chromosome 3 required for mitotic stability [22]. A major feature of this locus is a 16 kb GC-rich transcriptional 'strand-switch' domain composed predominantly of degenerate retroelements that separates two large directional gene clusters that are transcribed towards the telomeres. We proposed that this type of organization could serve as a model for centromeric DNA. The fragmented nature of the *T. cruzi *genome dataset [21] has negated testing of this hypothesis. Here, we demonstrate that an analogous region of chromosome 1 is required for mitotic stability and that the locations of these putative centromeres on both chromosomes coincide with sites of etoposide-mediated Topo-II cleavage. Furthermore, we show that Topo-II activity on *T. brucei *chromosomes also localizes to regions between directional gene clusters that contain degenerate retroelements, and additionally a domain of repetitive DNA.

## Results

### Similarity between the regions of *T. cruzi *chromosomes 1 and 3 required for mitotic stability

**Figure 1:**
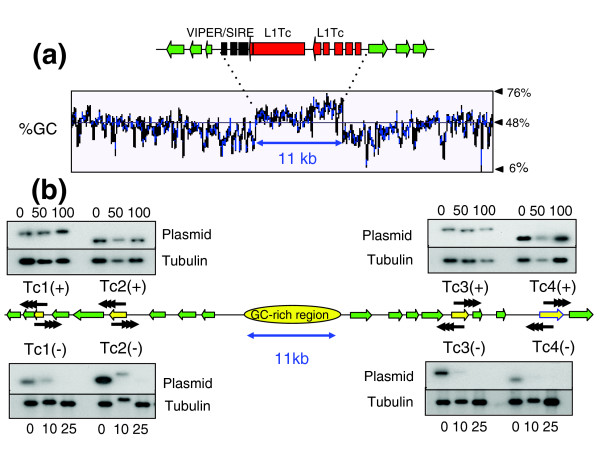
Functional mapping of the putative centromere on *T. cruzi *chromosome 1. **(a) **Organization of the GC-rich strand-switch region. Green arrows identify ORFs in the polycistronic gene clusters and the implied direction of transcription. The degenerate retrotransposon-like VIPER/SIRE element (black) and L1Tc autonomous retroelements (red) are indicated. The %GC content was determined by the Artemis 7 program [38]. **(b) **Mitotic stability of truncated chromosomes. Sequences used for fragmentation (Tc1-Tc4) are indicated by yellow arrows. Vectors were targeted in both directions (+/-), with black arrowheads representing the positions and orientations of *de novo *telomeres after fragmentation (see Additional data files 1-3 for further details). Clones with truncated chromosomes were grown in the absence of G418 for the generations indicated above or below the corresponding track. Genomic DNA was *Sca*I digested, Southern blotted, probed with plasmid DNA, then re-hybridized with β-tubulin as a loading control.

Chromosome 1 occurs as 0.51 Mb and 1.2 Mb homologues in the *T. cruzi *genome reference clone CL Brener. Because of the hybrid origin of this clone and the presence of extensive heterozygosity, it has not been possible to assemble fully contiguous sequences for the chromosomes of this parasite [21]. However, we have now identified a 300 kb contig, derived from chromosome 1, that contains an 11 kb GC-rich strand-switch domain composed mainly of degenerate retroelements, including a composite vestigial interposed repetitive retroelement/short interspersed repetitive element (VIPER/SIRE) and degenerate non-long terminal repeat (non-LTR) retrotransposon sequences (Figure 1a). This has remarkable organizational similarity to the putative centromeric region of chromosome 3 [22]. To determine if this domain is also required for mitotic stability, we used telomere-associated chromosome fragmentation to generate a series of cloned cell lines containing truncated versions of chromosome 1 (see Additional data files 1-3 for more details on these procedures). CL Brener displays allelic variation of 3% to 5% [24]. Primers used to amplify targeting fragments were based on sequence from the 0.51 Mb chromosome and most of the truncations arose from integration into this homologue. Analysis of mitotic stability focused on these (Figure 1b).

Clones containing truncations of the 0.51 Mb homologue were cultured in the absence of G418 (truncated chromosomes contain a *neo*^*r *^gene and associated plasmid DNA backbone [22]). Genomic DNA was assessed at various time points to determine the level of each truncated chromosome (Figure 1b). All four shortened chromosomes that retained the GC-rich strand-switch domain (+) were found to be maintained for more than 100 generations (5 months) in the absence of the selective drug. In contrast, chromosomes lacking this domain (-) were unstable and disappeared in 10-25 generations. Therefore, in both chromosome 1 and 3 of *T. cruzi*, we have now shown that the region required for mitotic stability centers on a GC-rich strand-switch domain composed predominantly of degenerate retrotransposons.

### Etoposide-mediated Topo-II cleavage sites in *T. cruzi *chromosomes are associated with regions required for mitotic stability

**Figure 2:**
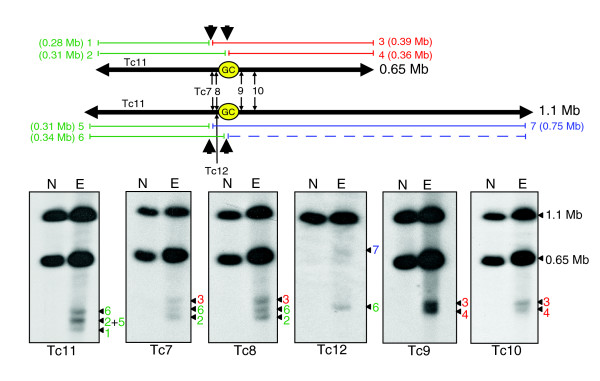
Etoposide-mediated cleavage sites in *T. cruzi *chromosome 3. Epimastigotes were treated with 1 mM etoposide for 6 h and chromosomal DNA fractionated by CHEFE and assessed by Southern analysis. Probe Tc12 is specific to the larger homologue (see Materials and methods). Lane N, non-treated parasites; lane E, etoposide-treated. The schematic shows both chromosome 3 homologues, location of the 16 kb GC-rich strand-switch domain (GC), positions of the probes and predicted locations of the major Topo-II cleavage sites (large black arrowheads). The fragments generated (1-7), and their sizes and inferred positions on the chromosomes are shown in red, green and blue. With the exception of probe Tc12, fragments derived from the right arm of the 1.1 Mb homologue (blue) cannot be detected, due to co-migration with the cross-hybridizing 0.65 Mb homologue.

In CL Brener, chromosome 3 occurs as homologues of 0.65 and 1.1 Mb (Figure 2). Most of this difference is due to a 0.40 Mb insertion in the right arm of the larger homologue, although the left arm is also 30 kb longer. Previously, we delineated the determinants of mitotic stability on chromosome 3 [22]. To provide independent evidence that this region has centromeric properties, we have now used Topo-II activity as a biochemical marker for active centromeres [15-18]. The procedure involved etoposide treatment of epimastigotes to promote double-stranded cleavage at the sites of Topo-II accumulation, isolation of chromosomal DNA and Southern analysis following fractionation by contour-clamped homogenous electric field gel electrophoresis (CHEFE).

We identified two major etoposide-mediated cleavage sites in the vicinity of the GC-rich strand-switch domain of *T. cruzi *chromosome 3 (Figure 2). Bands of 0.39, 0.34 and 0.31 Mb were detected with probes Tc7 and Tc8, sequences from the left arm of the chromosome, 20 and 10 kb respectively, from the strand-switch domain. The Tc11 probe, from a gene array closer to the left telomere, identified products of 0.34, 0.31 and 0.28 Mb. The 0.28 Mb fragment (band 1), which was not detected with probes Tc7 and Tc8, allows tentative location of one cleavage site to a region 30 kb from the strand-switch domain on the smaller chromosome (Figure 2). Cleavage at the corresponding site on the 1.1 Mb chromosome should generate a 0.31 Mb product (band 5), since the left arm of this homologue is 30 kb longer. The 0.34 Mb product (band 6) in the Tc11 autoradiograph can be inferred to arise from a second cleavage site located within the strand-switch domain of the 1.1 Mb chromosome. This band hybridizes to probe Tc12, a sequence unique to the larger homologue. Cleavage at this site in the 0.65 Mb chromosome should generate a 0.31 Mb fragment (band 2, Tc7, Tc8 and Tc11). In the Tc11 autoradiograph, this fragment co-migrates with band 5, a cleavage product derived from the larger homologue. Probes Tc9 and Tc10, from the right arm of chromosome 3 (Figure 2), hybridized to cleavage products of 0.39 and 0.36 Mb (bands 3 and 4). Cleavage of the smaller homologue, at the sites predicted above, should generate these products. The corresponding cleavage of the 1.1 Mb chromosome would produce a band masked by the hybridization signal of the intact smaller homologue. A product of this size was detected with homologue-specific probe Tc12 (band 7).

**Figure 3:**
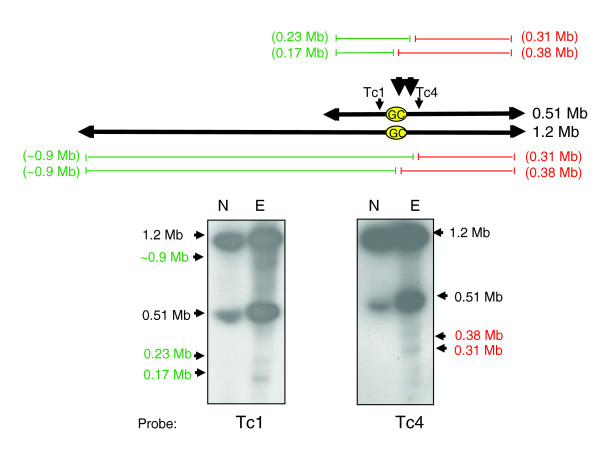
Mapping of etoposide-mediated Topo-II cleavage sites in *T. cruzi *chromosome 1. Epimastigotes were treated with 1 mM etoposide for 6 h and chromosomal DNA fractionated by CHEFE and assessed by Southern hybridization. Probes Tc1 and Tc4 were used (Additional data file 5). Large black arrowheads identify the predicted locations of Topo-II activity adjacent to the GC-rich strand-switch domain (yellow oval). The cleavage fragments are identified in red and green. Lane N, non-treated parasites; lane E, etoposide-treated.

Together, these data indicate the presence of two major sites of Topo-II accumulation on chromosome 3, one located within the strand-switch region and one approximately 30 kb downstream. Therefore, functional [22] and biochemical mapping now provide independent evidence of an active centromere at this locus. We also investigated if etoposide treatment resulted in lesions close to the GC-rich strand-switch region of chromosome 1 (Figure 3). The data confirm that the presence of two major sites of Topo-II activity on chromosome 1, situated close to the strand-switch domain, within the region required for mitotic stability.

### Synteny in the location of Topo-II activity on *T. brucei *chromosome 1 and *T. cruzi *chromosome 3

**Figure 4:**
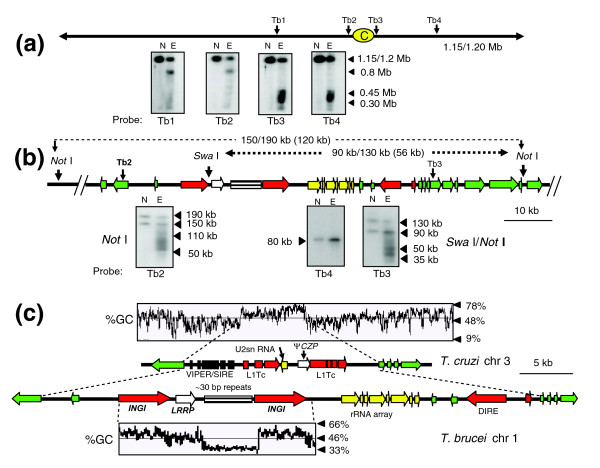
Etoposide-mediated cleavage sites on *T. brucei *chromosome 1. **(a) **Procyclics were treated with 500 μM etoposide for 1 h and chromosomal DNA fractionated by CHEFE. Hybridization was carried out with probes Tb1-Tb4. Their positions and the location of the strand-switch domain (yellow oval) are shown. Lane N, non-treated parasites; lane E, etoposide-treated. **(b) **Fine-mapping of cleavage sites. Chromosomal DNA from treated/non-treated parasites was immobilized in agar blocks, restriction digested and fractionated by CHEFE. Fragment sizes are shown above the schematic, with their predicted sizes (GeneDB) in parentheses. Black triangles identify the fragments and cleavage products on the relevant autoradiographs. As control, blots were re-hybridized with probe Tb4, from a gene 150 kb upstream of the putative centromere. **(c) **Comparison of the *T. cruzi *chromosome 3 centromeric domain with the syntenic region of *T. brucei *chromosome 1. In the *T. cruzi *chromosome, the degenerate VIPER/SIRE element and L1Tc retroelements are indicated, together with a truncated cruzipain pseudogene (*ψCZP*) and an U2snRNA gene. The corresponding region in *T. brucei *chromosome 1 contains 2 *INGI *retrotransposons and 1 DIRE. The locations of a leucine rich repeat protein gene (*LRRP*), a rRNA gene array and a 5.5 kb array of approximately 30 bp repeats are shown. Green arrows indicate putative ORFs and the implied direction of transcription. The dashed lines between the *T. cruzi *and *T. brucei *maps identify the equivalent positions of the first ORFs of the conserved directional gene clusters.

Despite a completed genome sequence, there are no experimental data on centromere location in *T. brucei*. To address this, we treated procyclic parasites with etoposide and mapped the sites of Topo-II activity. With chromosome 1 (homologues of 1.15 and 1.2 Mb), probes from the left arm of the chromosome (Tb1 and Tb2) hybridized to a major cleavage product of 0.8 Mb, whereas those from the right arm (Tb3 and Tb4) identified a smear ranging from 0.3-0.45 Mb (Figure 4a). These data localize etoposide-mediated cleavage of chromosome 1 to the region between probes Tb2 and Tb3. The more extensive smearing of products from the right arm of the chromosome further suggests that the main size differences between the homologues result from additional sequences in this arm of the chromosome. In *T. brucei*, most differences between homologues are restricted to the subtelomeric regions.

To assess the extent of the Topo-II 'footprint' on this chromosome, we analyzed restriction digested genomic DNA. With DNA from non-treated parasites, *Not*I digestion generated two bands detectable with probe Tb2, indicative of differences in the lengths of the corresponding regions on each homologue (Figure 4b). Furthermore, these regions were larger than inferred from the genome sequence [23]. Bands of 150 and 190 kb were generated rather than the predicted 120 kb. Similarly, *Swa*I/*Not*I digestion produced fragments of 90 and 130 kb, instead of the expected 56 kb. With DNA from etoposide-treated parasites, we observed a series of major cleavage products and a smear that stretched >60 kb (Figure 4b). Cleavage sites could not be accurately mapped onto the chromosome because of the heterogeneity between homologues, and possible gaps in sequence assembly. Nevertheless, it is implicit that Topo-II activity is regional, confined to the sequence between probes Tb2 and Tb3 (approximately 85 kb/120 kb, depending on the homologue), and exhibits significant site-specificity within this region. When blots were hybridized with probe Tb4, from an open reading frame (ORF) 150 kb upstream, there was minimal etoposide-mediated cleavage.

Intriguingly, the location of Topo-II activity on *T. brucei *chromosome 1 is syntenic with the region of etoposide-mediated cleavage on *T. cruzi *chromosome 3 (Figure 2), which is also required for mitotic stability [22]. This suggests that centromere location on these chromosomes has been conserved since species divergence, more than 200 million years ago. In *T. brucei*, this region encompasses a transcriptional strand-switch domain containing two full-length *INGI *retrotransposon-like elements closely linked to a degenerated *INGI*/L1Tc-related element (DIRE), and a short array of ribosomal RNA genes (Figure 4c). A major difference between the domains is the presence in the *T. brucei *chromosome of a 5.5 kb element of degenerate AT-rich repeats of approximately 30 bp. The analogous region of *T. cruzi *chromosome 3 lacks any kind of repetitive array. In the case of the putative centromere of *T. cruzi *chromosome 1 (Figure 1), the corresponding region is located on *T. brucei *chromosome 11, associated with a break in synteny.

### Repetitive arrays are a feature of Topo-II cleavage sites in *T. brucei *chromosomes

**Figure 5:**
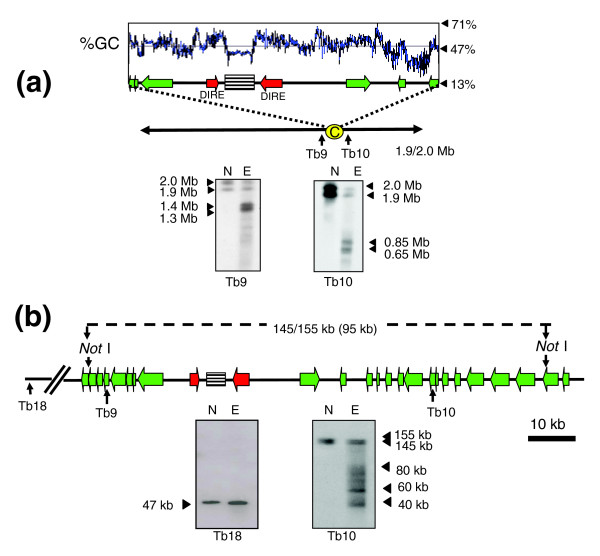
Etoposide-mediated cleavage sites on *T. brucei *chromosome 4. **(a) **Procyclics were treated with 500 μM etoposide for 1 h and chromosomal DNA fractionated by CHEFE and Southern blotted. Red arrows identify DIREs and green arrows indicate putative ORFs and the implied direction of transcription. The location of the AT-rich repeat array is highlighted (striped box). The positions of probes and location of the putative centromeric region (yellow oval) are indicated. Lane N, non-treated parasites; lane E, etoposide-treated. **(b) **Fine mapping of cleavage sites. *Not*I digested DNA was fractionated by CHEFE as in Figure 4b and Southern blotted. Fragment sizes are shown above the schematic, with their predicted sizes (GeneDB) in parentheses. Black triangles identify these fragments on the autoradiograph and show the major cleavage products. As control, the membrane was hybridized with probe Tb18, from a gene 500 kb downstream of the putative centromere.

With *T. brucei *chromosome 4 (homologues of 1.9 and 2 Mb), the major products generated by etoposide treatment were doublets of 1.3/1.4 Mb (probe Tb9) and 0.65/0.85 Mb (probe Tb10) (Figure 5a). The ends of chromosome 4 have not been fully assembled [23] and it was not possible to accurately map the cleavage sites on the basis of product size. However, it can be inferred from the hybridization patterns that the sites are located between the ORFs from which probes Tb9 and Tb10 were derived (Figure 5a). This sequence contains a domain that separates directional gene clusters, with head-to-head DIREs located either side of a 3.5 kb AT-rich element made up of 149 bp repeats. There are no other repetitive arrays elsewhere on chromosome 4.

The extent of Topo-II activity in this region was investigated further by Southern analysis of *Not*I digested DNA. Based on the genome sequence, the major cleavage sites were expected to be within a 95 kb fragment. However, *Not*I digestion generated a doublet of 145/155 kb that covers this region (probe Tb10; Figure 5). As with chromosome 1, this could reflect heterogeneity between chromosome homologues and possible gaps in sequence assembly. In the track containing DNA from etoposide-treated cells, four major products of 40-80 kb were identified on a background smear. Precise localization of the corresponding sites on the genome map is complicated by the issues discussed above. Nevertheless, it is implicit from the data (Figure 5a, b) that Topo-II activity on chromosome 4 is concentrated in this region, which contains an array of AT-rich repeats, similar to the putative centromeric region of chromosome 1. Using a probe 500 kb distant from this domain (Tb18), we detected minimal etoposide-mediated cleavage.

**Figure 6:**
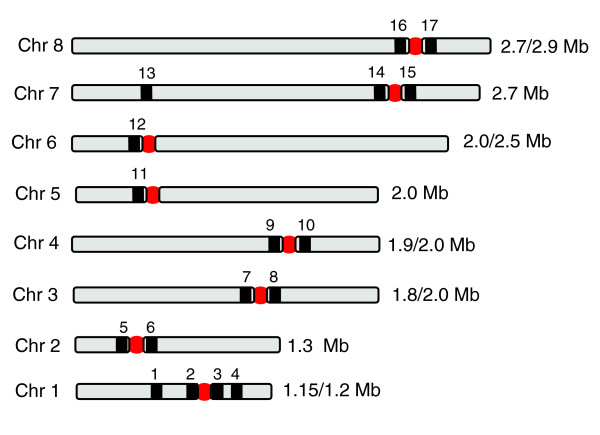
Etoposide-mediated Topo-II cleavage sites (red circles) on *T. brucei *(strain 927) chromosomes 1-8. The locations of the probes (Tb1-17) are identified by black bars. Details on the AT-rich arrays are given in Figure 7 and Table 1 and the experimental results are shown in Additional data file 4.

**Figure 7:**
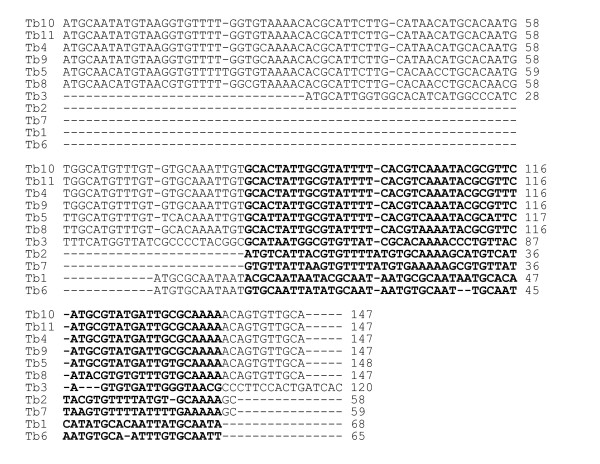
Alignment of repeat array consensus sequences on each *T. brucei *chromosome, determined by the program Tandem Repeats Finder [39]. The nucleotides marked in bold correspond to two copies of the array (approximately 30 bp) in chromosomes 1, 2, 6 and 7.

To assess if repeat arrays are a conserved feature of Topo-II accumulation sites, we delineated these regions in chromosomes 1-8 (the results are shown in Additional data file 4 and summarized in Figure 6). Etoposide-mediated cleavage sites could be mapped to regions between specific gene probes, or inferred from the sizes of the cleavage products. In each case, Topo-II binding was closely associated with regions that separate directional gene clusters and contain at least one DIRE or *INGI *retroelementand an array of AT-rich repeats. The arrays are restricted to a single site on each chromosome. They typically range from 2-8 kb, although on chromosome 3 the estimate is 30 kb, and others (chromosomes 6 and 8) remain to be fully sequenced (GeneDB). Contiguous sequences for chromosomes 9, 10 and 11 have yet to be assembled and we did not attempt their analysis. However, sequences similar to the AT-rich repeats have been assigned to these chromosomes. The consensus repeat sequences for each chromosome and a summary of their properties are given in Figure 7 and Table 1, respectively.

**Table 1:** Summary of information available (GeneDB) on the AT-rich repeat domains

Chromosome number	Total size of repeat domain (kb)	Unit repeat size (bp)	AT content (%)	AT-rich array position orientated by flanking ORFs
1	5.5	~30	66	Tb927.1.3670 - Tb927.1.3750
2	8.0	29	66	Tb927.2.1470 - Tb927.2.1560
3	Unknown*	120	49	Tb927.3.3410 - Tb927.3.3430
4	3.5	147	61	Tb927.4.3740 - Tb927.4.3750
5	2.3	148	62	Tb927.5.600 - Tb927.5.610
6	Unknown^†^	~30	71	Tb927.6.180 - Tb927.6.190
7	3.0	29	75	Tb927.7.6860 - Tb927.7.6870
8	Unknown^‡^	147	59	Tb927.8.7740 - Tb927.8.7750
9	Unknown^§^	147	60	Incomplete assembly
10	Unknown^¶^	147	61	Incomplete assembly
11	Unknown^¥^	147	61	Incomplete assembly

The AT-rich repeat domains are associated with the sites of etoposide-mediated Topo-II cleavage on *T. brucei *chromosomes 1-8 (Additional data file 4 and summarized in Figure 6). The flanking ORFs are those immediately adjacent to the repeat array. *BAC clone RPCI93-3K10 contains a large number of 120 bp repeats that span up to 30 kb (GeneDB). This region has not yet been closed. ^†^The repeat array contains a gap of unknown size. ^‡^There are 1.5 copies of this repeat next to a gap in the chromosome 8 sequence at position 2,233,104 bp. The unresolved array is estimated to be 5-9 kb (GeneDB). ^§^Several chromosome 9 reads (approximately 700 bp) contain these repeats (for example, tryp_IXb-344h12.p1c, GeneDB). ^¶^A 10 kb contig tryp_X-275g09.p1c (Tb10.v4.0130) and several chromosome 10 reads (approximately 700 bp) contain these repeats (for example, tryp_X-429d08.q1c, GeneDB). ^¥^A 14 kb contig tryp_XI-974h12.q1k (Tb11.1750) and several chromosome 11 reads (approximately 900 bp) contain these repeats (for example, tryp_XI-991b09.q1k, GeneDB).

Based on available sequence (GeneDB), the AT-rich repeats fall into four classes. In the largest, chromosomes 4, 5, 8, 9, 10 and 11, they are organized in units of approximately 147 bp and share >90% identity. These units have a complex structure built from degenerate sub-repeats of approximately 48 and 30 bp. Where these arrays have been fully sequenced, they do not display a gradient of divergence moving from the centre towards the edge, unlike the α-satellite repeats in human centromeric DNA [5]. In two of the other groupings, chromosomes 2/7 and chromosomes 1/6, the arrays are made up of repeats of approximately 30 bp, which share 83% and 76% identity, respectively. The array in chromosome 3 is distinctive; it is organized in units of 120 bp, with an AT content of only 49%. Despite this, it is related to the other repeats, for example, sharing 53% identity with that of chromosome 4. We also noted that the repeat regions were adjacent to arrays of rRNA genes in chromosomes 1, 2, 3, 6 and 7, although the significance of this is unknown.

### *T. brucei *intermediate and minichromosomes are refractory to etoposide-mediated cleavage

**Figure 8:**
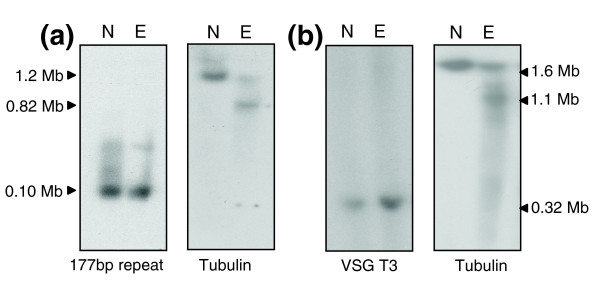
*T. brucei ***(a) **minichromosomes and **(b) **intermediate chromosomes are resistant to etoposide-mediated cleavage. (a) Procyclics (927 strain) were treated with 500 μM etoposide for 3 h and chromosomal DNA separated by CHEFE. Southern blots were hybridized with the 177 bp repeat probe or α-tubulin. (b) Bloodstream form *T. brucei *(427 strain) were treated with 25 μM etoposide for 1 h and chromosomal DNA analyzed as above. Blots were hybridized with the intermediate chromosome-specific probe T3. In strain 427, chromosome 1 is larger than in strain 927, as is the major cleavage product identified with the α-tubulin probe. Lane N, non-treated parasites; lane E, etoposide-treated.

In addition to 11 'conventional' chromosomes, *T. brucei *also contains approximately 100 minichromosomes and several intermediate-sized chromosomes [25]. We investigated if these were susceptible to etoposide-mediated cleavage. For the minichromosomes, we used the 177 bp repeat as a probe. This element is also present on intermediate-sized chromosomes, but in considerably fewer copies (Figure 8a). In contrast to chromosome 1, which was analyzed in parallel using an α-tubulin probe, we could detect no evidence of minichromosome cleavage, even when etoposide treatment was extended for three hours. To assess the intermediate chromosomes, we used bloodstream forms of the *T. brucei *427 strain and the T3 *VSG *probe. This fragment hybridizes to a 0.32 Mb intermediate-sized chromosome in strain 427 (D Horn, personal communication). Generally, we observed that cleavage of the mega-based sized chromosomes in bloodstream form parasites required lower drug concentrations and shorter incubation periods than in procyclics. Under conditions in which there was significant site-specific cleavage of chromosome 1, we could detect no evidence for etoposide-mediated cleavage of the 0.32 Mb chromosome (Figure 8b). It can be inferred, therefore, that Topo-II does not undergo site-specific accumulation on the intermediate and minichromosomes.

## Discussion

With the completion of the trypanosome genome projects, the lack of information on the location of centromeric DNA is the major remaining gap in our understanding of chromosome organization. Here, we have exploited the genome sequence data and a combination of genetic and biochemical techniques to address this question. In the case of *T. cruzi*, centromere location was mapped using two independent approaches. The first, telomere-associated fragmentation, delineated the region required for the mitotic stability of chromosome 1 to a 40 kb sequence with striking organizational similarity to the putative centromeric region of chromosome 3 [22]. Consistent with this, we mapped Topo-II activity to these same regions on both chromosomes. In mammalian cells, etoposide-mediated cleavage sites are biochemical markers of centromeric DNA [12,15-18]. As cells enter mitosis, sister chromatids remain attached, partly through strand catenation at centromeres [8]. Centromeric accumulation of Topo-II at this stage of the cell cycle acts to regulate sister chromatid cohesion and enzyme activity is essential for ordered segregation [9].

On the basis of these two independent approaches applied to two separate chromosomes, we now suggest a paradigm for *T. cruzi *centromeric DNA; GC-rich strand-switch domains composed predominantly of degenerate retrotransposons. This latter feature is shared to an extent with centromeres of higher eukaryotes, where transposable elements have been suggested to have important roles in centromere function, including mediating heterochromatin formation [26]. In human cells, functionally active and genetically stable neocentromeres can also form in euchromatic regions of chromosomes that lack repetitive arrays but are rich in LINE non-LTR retrotransposons [27]. By analogy, retrotransposons could have a role in some aspect of centromere function in *T. cruzi*. Alternatively, centromeric domains may simply provide a genomic niche that favors their integration and retention [28]. The regions that separate directional gene clusters are one of the few areas on *T. cruzi *chromosomes not transcribed constitutively. However, this itself cannot be a determinant of centromere location, since parasite chromosomes typically contain several such regions [21]. Only a sub-set of the *T. cruzi *strand-switch domains so far assembled have an organization similar to those identified on chromosomes 1 and 3. We therefore propose that only one strand-switch domain per chromosome will be found to have this type of configuration, a hypothesis that will be testable when a more complete version of the *T. cruzi *genome becomes available.

In *T. cruzi *chromosome 3, we detected two major Topo-II cleavage sites, one within the strand-switch region and the other 30 kb distant (Figure 2). Two cleavage sites were also detected in the region required for mitotic stability of chromosome 1 (Figure 1). These patterns could correlate with the functional boundary of a 'centromeric domain' or merely reflect other aspects of higher-order chromatin structure formed in the neighborhood of an active centromere. In human chromosomes, although etoposide-mediated Topo-II cleavage is confined to the arrays of α-satellite DNA, enzyme binding appears to be dependent on structural features associated with chromatin, rather than DNA sequence [17].

Interest in trypanosomatid Topo-II has stemmed mainly from its potential as a drug target [29] and its role in replication of kinetoplast DNA minicircles [30,31]. Trypanosomes have distinct classes of mitochondrial and nuclear Topo-II. In *T. brucei*, there are two genes for nuclear isoforms, *TbTOP2α *and *TbTOP2β*, although the latter may be a pseudogene. TbTOP2α is essential, with RNAi-mediated knockdown causing growth arrest and abnormalities in nuclear morphology consistent with mis-segregation [32]. We found Topo-II activity to be a regional phenomenon in *T. brucei *chromosomes, concentrated at single sites located between directional gene clusters. These zones contain at least one DIRE/*INGI *retrotransposon and an array of AT-rich repeats, conserved to varying degrees between chromosomes. We propose that this type of organization is a central feature of centromeric DNA in *T. brucei*. The major difference in the putative centromeric regions of *T. cruzi *and *T. brucei *is the presence of repetitive arrays in the latter. Centromeric repeats in other eukaryotes display considerable sequence variation, even between closely related species. In *T. brucei*, these arrays exhibit a range of intra-chromosomal divergence and appear to be dynamic in terms of recombination, with evidence that active processes operate to generate both sequence homogenization and unequal crossover. Complete sequencing of the arrays on each chromosome may reveal more of the mechanisms involved. Similarly, resolution of discrepancies between sequence data and restriction analysis, such as those in the centromeric domains of chromosomes 1 and 4 (Figures 4 and 5), should facilitate more detailed functional mapping of these regions.

In terms of centromere organization, a major question remains: were the repeat elements lost by *T. cruzi *or did they arise only in *T. brucei*? *Leishmania *diverged early in trypanosomatid evolution, followed by a later split in the *Trypanosoma *lineage. Evidence from genome analysis suggests that the progenitor species had a karyotype more analogous to *T. cruzi *and *Leishmania major *(approximately 30 and 36 chromosome pairs, respectively) [21], both of which lack this class of repeat element. Therefore, the repetitive DNA probably became a feature of *T. brucei *centromeres after their divergence from *T. cruzi*. Retrotransposons have been proposed as templates for the evolution of centromeric repeats in higher eukaryotes [26]. In this context, one explanation for the presence of repetitive DNA at *T. brucei *centromeres could be that local chromatin provided an environment that supported its expansion and propagation. It could be feasible, using transfection-based approaches, to test if these arrays confer mitotic stability. However, in other eukaryotes, the fact that centromere location is determined epigenetically has often complicated the interpretation of such experiments.

We could detect no significant etoposide-mediated cleavage of *T. brucei *intermediate or minichromosomes (Figure 8), implying that site-specific Topo-II activity does not have a role in their segregation. Any mechanism for minichromosome segregation must account for the high fidelity of the process and the observation that the number of minichromosomes exceeds the number of microtubule spindles. The 'lateral-stacking model' [25] proposes that replicated minichromosomes attach laterally to anti-parallel microtubules, with directional poleward movement following chromatid separation. It is envisaged that several replicated minichromosomes could attach to each microtubule pair. This model is centromere-independent and lacks a specific requirement for Topo-II accumulation at single sites on the minichromosome, or the intermediate-sized chromosomes, if an analogous mechanism is involved. Similar to the situation with the intermediate and minichromosomes, we could not detect site-specific etoposide-mediated cleavage of *L. major *chromosomes (data not shown). In this instance, however, it could be that the drug cannot access the parasite nucleus.

The evidence that we report here provides a basis for identifying other determinants of centromere function. This is important, since several observations hint that aspects of segregation in trypanosomes differ from the mammalian host. The number of kinetochores appears to be less than the number of chromosomes [33,34], trypanosomes lack obvious equivalents of the 'core' centromeric proteins [1] and few of the other proteins involved in kinetochore assembly/function have been conserved [23]. It may be that segregation in trypanosomes is more streamlined than in other organisms, or that the proteins involved are highly divergent. Whatever the reason, resolution of these issues should provide new insights into the evolution of this complex process and could lead to the identification of novel parasite proteins that might serve as targets for chemotherapy.

## Conclusion

We report the first experimental evidence on the location of centromeres in both *T. cruzi *and *T. brucei*. Centromeric DNA in these primitive eukaryotes is associated with regions between directional gene clusters that contain degenerate retroelements. In *T. brucei*, but not *T. cruzi*, these loci also contain arrays of AT-rich repeats. These findings provide a framework for investigating chromosome segregation in a more systematic manner. Until now, progress in this area has been extremely limited.

## Materials and methods

### Parasites and DNA preparation

*T. cruzi *epimastigotes (CL Brener) were cultured as described [22]. *T. brucei *procyclics (genome project TREU 927/4 strain) were grown at 27°C in SDM-79 medium [35] and bloodstream forms (427 strain) at 37°C in a 5% CO_2 _atmosphere in modified Iscove's medium [36]. Genomic DNA was extracted using DNeasy tissue mini-kits (Qiagen, Venlo, Netherlands). Intact chromosomes extracted by an agarose-embedding technique [22] were separated with a CHEF Mapper System (Bio-Rad, Hercules, CA, USA). For *in situ *restriction digestion, agarose blocks were incubated in 1 mM phenylmethanesulphonylfluoride (PMSF) at 25°C for 1 h, washed in TE buffer (10 mM Tris-Cl, 1 mM EDTA, pH7.4) and incubated with enzyme for 24 h.

### Analysis of transformants

For targeted chromosome fragmentation, we used vector pTEX-CF [22]. Briefly, targeting fragments were amplified and inserted into the vector. Gel-purified linear DNA (1-2 μg) was then introduced into *T. cruzi *epimastigotes by electroporation [22]. Transformed cells were selected over 6-8 weeks in 100 μg ml^-1 ^G418, and cloned by limiting dilution. Clones were analyzed by Southern blotting of CHEFE-separated chromosomal DNA and by sequencing of transformant-specific PCR fragments [22]. Transformants containing truncated forms of chromosome 1 were continuously cultured in the presence or absence of G418, and DNA isolated at various time points. Typically, mitotic stability was assessed by Southern hybridization using plasmid DNA as a probe for truncated chromosomes. Results were confirmed by CHEFE.

### Analysis of etoposide-treated cells

Logarithmically growing *T. cruzi *epimastigotes and *T. brucei *procyclics were treated with DMSO-solubilized etoposide (Sigma, St Louis, MO, USA). Drug dose and incubation times optimal for generating specific cleavage fragments were determined empirically for each parasite. Chromosomal DNA was then isolated and fractionated by CHEFE, or genomic DNA prepared. DNA probes were generated by PCR. For *T. cruzi *chromosome 3, we amplified a 1.1 Mb homologue-specific 290 bp fragment (probe Tc12; Figure 2) using primers ACCATGTCCAAATTGATCTGCAAATCA and TAGAAGCATTAAATCCCAGCATCAGAC. For *T. brucei *chromosome 6, we used an intergenic probe (Tb12), which was amplified with primers TCTTGATAGGCGCATGTGCATGTAACC and CGATGGCGCAAGCAGATAGTTGTTACC. In the case of the *T. brucei *177 bp repeat [37], a probe was generated using primers ATTAAACAATGCGCAGTTAACG and GTGTATAATAGCGTTAACTGCG. Other probes are described in Additional data file 5.

## Additional data files

The following additional data are available with the online version of this paper. Additional data file 1 outlines the strategy for telomere-associated chromosome fragmentation of the small homologue of *T. cruzi *chromosome 1. The schematic of the 0.51 Mb chromosome shows the position of the 11 kb GC-rich strand-switch domain (yellow oval). The expanded section (70 kb) contains the four ORFs used for chromosome fragmentation (Tc1-Tc4; Additional data file 5). The implied direction of polycistronic transcription is indicated. A 0.9 kb DNA fragment from ORF Tc1 was cloned into the pTEX-CF vector [22]. This was linearized so that the targeting fragment was at one end and telomeric sequences at the other. Plasmid DNA within the vector is marked in red. Site-specific integration (crossed lines) results in the deletion of approximately 100 kb of DNA between the target sequence and the telomere (Additional data file 2). Telomeric sequences supplied by the vector are shown as horizontal arrowheads. Expression of *neo*^*r *^is under control of the rDNA promoter (flagged). Additional data file 2 shows the generation of a truncated version of the small homologue of *T. cruzi *chromosome 1. The 11 kb GC-rich strand-switch domains are shown (yellow oval). Arrows (Tc1-Tc4) indicate positions of the target sequences used for fragmentation (Additional data file 5). Parasites were transfected with linearized vector DNA and transformants selected with G418 [22] (see Materials and methods). Chromosomal DNA from wild-type (WT) and transfected (T) parasites was separated by CHEFE with *S. cerevisiae *markers (M) and analyzed by Southern blotting using plasmid, Tc5 and Tc6 radiolabelled probes. Autoradiographs are shown, together with an ethidium bromide (EtBr) stained gel, overexposed to show the truncated chromosome. In the example shown, integration at the Tc1 locus of the 0.51 Mb chromosome, in the direction of polycistronic transcription, results in deletion of approximately 100 kb of DNA between the target sequence and the telomere. Hybridization identifies the larger (1.2 Mb) and smaller homologues (0.51 Mb) and the truncated product (0.40 Mb). Plasmid DNA is incorporated into truncated chromosomes as part of the integration process (Additional data file 1). Additional data file 3 shows how chromosome fragmentation demonstrates that the major size difference between the *T. cruzi *chromosome 1 homologues is the result of the insertion/deletion of 0.7 Mb of DNA into/from the left arm of the chromosome. **(a) **Schematic showing the 0.51 Mb and 1.2 Mb homologues and their respective truncated products C1 (0.2 Mb) and C2 (0.9 Mb), with the locations of probes Tc2-Tc5 (Additional data file 5). **(b) **Autoradiograph illustrating the deletion of the right arms of each chromosome homologue following integration of the fragmentation vector at ORF Tc4. Two clones were isolated after transfection and chromosomal DNA from wild-type (WT) and both clones (C1 and C2) were separated by CHEFE and analyzed by Southern blotting using radiolabeled probe Tc4. Hybridization identifies the larger homologue (1.2 Mb), the smaller homologue (0.51 Mb) and their respective truncated products (0.9 Mb and 0.2 Mb). We had previously shown that probe Tc5 is located a similar distance (approximately 50 kb) from the end of both homologues [22]. Additional data file 4 shows mapping of etoposide-mediated Topo-II cleavage sites in *T. brucei *chromosomes 1-8. *T. brucei *procyclic cultures were treated with 500 μM etoposide and chromosomal DNA isolated and fractionated using a Bio-Rad CHEF Mapper system. Membranes were hybridized with probes Tb1-Tb17, the GeneDB systemic names of which are shown in Additional data file 5. The positions of probes (arrows) and locations of the putative centromeric regions (yellow ovals) are indicated. Red arrows indicate *INGI*/DIRE retroelements and green arrows identify putative ORFs and the implied direction of transcription. The locations of the AT-rich repeat arrays are highlighted (striped box) and the %GC contents across the regions are shown. GeneDB systemic names of ORFs adjacent to the arrays are given and allow the repeat arrays to be mapped onto the chromosomal contigs. Lane N, non-treated parasites; lane E, etoposide-treated. Chromosome sizes were calculated using *S. cerevisiae *(0.2-2.2 Mb) and *Hansenula wingei *(1.0-3.1 Mb) chromosome markers (Bio-Rad). In most cases, sequence contigs do not extend to the ends of chromosomes. Gaps in the sequence of AT-rich arrays in chromosomes 3, 6 and 8 are indicated by asterixes and double slashes. We have also found, using long-range restriction mapping (Figures 4b and 5b), that the 'centromeric-domains' of chromosomes 1 and 4 are larger than predicted by the genome sequence. Additional data file 5 is a list of *T. cruzi *(Tc1-12) and *T. brucei *(Tb1-18) probes used in this study, together with their GeneDB systemic names.

## Supplementary Material

Additional data file 1 The schematic of the 0.51 Mb chromosome shows the position of the 11 kb GC-rich strand-switch domain (yellow oval). The expanded section (70 kb) contains the four ORFs used for chromosome fragmentation (Tc1-Tc4; Additional data file 5). The implied direction of polycistronic transcription is indicated. A 0.9 kb DNA fragment from ORF Tc1 was cloned into the pTEX-CF vector [22]. This was linearized so that the targeting fragment was at one end and telomeric sequences at the other. Plasmid DNA within the vector is marked in red. Site-specific integration (crossed lines) results in the deletion of approximately 100 kb of DNA between the target sequence and the telomere (Additional data file 2). Telomeric sequences supplied by the vector are shown as horizontal arrowheads. Expression of *neo*^*r *^is under control of the rDNA promoter (flagged) Click here for file

Additional data file 2 The 11 kb GC-rich strand-switch domains are shown (yellow oval). Arrows (Tc1-Tc4) indicate positions of the target sequences used for fragmentation (Additional data file 5). Parasites were transfected with linearized vector DNA and transformants selected with G418 [22] (see Materials and methods). Chromosomal DNA from wild-type (WT) and transfected (T) parasites was separated by CHEFE with *S. cerevisiae *markers (M) and analyzed by Southern blotting using plasmid, Tc5 and Tc6 radiolabelled probes. Autoradiographs are shown, together with an ethidium bromide (EtBr) stained gel, overexposed to show the truncated chromosome. In the example shown, integration at the Tc1 locus of the 0.51 Mb chromosome, in the direction of polycistronic transcription, results in deletion of approximately 100 kb of DNA between the target sequence and the telomere. Hybridization identifies the larger (1.2 Mb) and smaller homologues (0.51 Mb) and the truncated product (0.40 Mb). Plasmid DNA is incorporated into truncated chromosomes as part of the integration process (Additional data file 1) Click here for file

Additional data file 3 **(a) **Schematic showing the 0.51 Mb and 1.2 Mb homologues and their respective truncated products C1 (0.2 Mb) and C2 (0.9 Mb), with the locations of probes Tc2-Tc5 (Additional data file 5). **(b) **Autoradiograph illustrating the deletion of the right arms of each chromosome homologue following integration of the fragmentation vector at ORF Tc4. Two clones were isolated after transfection and chromosomal DNA from wild-type (WT) and both clones (C1 and C2) were separated by CHEFE and analyzed by Southern blotting using radiolabeled probe Tc4. Hybridization identifies the larger homologue (1.2 Mb), the smaller homologue (0.51 Mb) and their respective truncated products (0.9 Mb and 0.2 Mb). We had previously shown that probe Tc5 is located a similar distance (approximately 50 kb) from the end of both homologues [22] Click here for file

Additional data file 4 *T. brucei *procyclic cultures were treated with 500 μM etoposide and chromosomal DNA isolated and fractionated using a Bio-Rad CHEF Mapper system. Membranes were hybridized with probes Tb1-Tb17, the GeneDB systemic names of which are shown in Additional data file 5. The positions of probes (arrows) and locations of the putative centromeric regions (yellow ovals) are indicated. Red arrows indicate *INGI*/DIRE retroelements and green arrows identify putative ORFs and the implied direction of transcription. The locations of the AT-rich repeat arrays are highlighted (striped box) and the %GC contents across the regions are shown. GeneDB systemic names of ORFs adjacent to the arrays are given and allow the repeat arrays to be mapped onto the chromosomal contigs. Lane N, non-treated parasites; lane E, etoposide-treated. Chromosome sizes were calculated using *S. cerevisiae *(0.2-2.2 Mb) and *Hansenula wingei *(1.0-3.1 Mb) chromosome markers (Bio-Rad). In most cases, sequence contigs do not extend to the ends of chromosomes. Gaps in the sequence of AT-rich arrays in chromosomes 3, 6 and 8 are indicated by asterixes and double slashes. We have also found, using long-range restriction mapping (Figures 4b and 5bB), that the 'centromeric-domains' of chromosomes 1 and 4 are larger than predicted by the genome sequence Click here for file

Additional data file 5 *T. cruzi *(Tc1-12) and *T. brucei *(Tb1-18) probes used in this study, together with their GeneDB systemic names Click here for file
