# Supplementary material for: Repetitive DNA is associated with centromeric domains in Trypanosoma brucei but not Trypanosoma cruzi
Source: Genome Biol. 2007 Mar 12;8(3):R37. doi: 10.1186/gb-2007-8-3-r37 (PMC1868937; doi:10.1186/gb-2007-8-3-r37)

Additional Data File 1.

Strategy for telomere-associated chromosome fragmentation of the small homologue of *T. cruzi* chromosome 1.

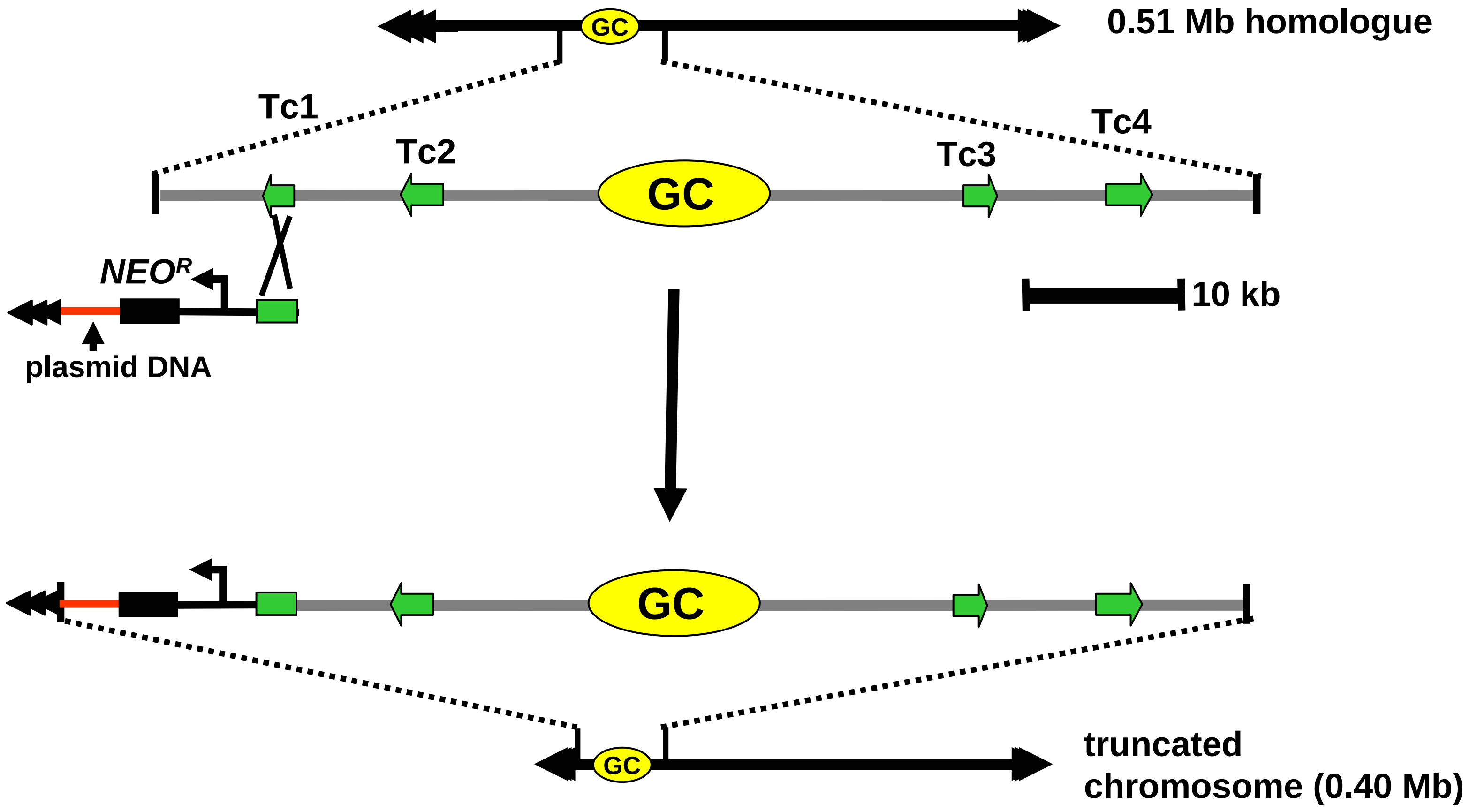

Supplement: Additional data file 1 — The schematic of the 0.51 Mb chromosome shows the position of the 11 kb GC-rich strand-switch domain (yellow oval). The expanded section (70 kb) contains the four ORFs used for chromosome fragmentation (Tc1-Tc4; Additional data file 5). The implied direction of polycistronic transcription is indicated. A 0.9 kb DNA fragment from ORF Tc1 was cloned into the pTEX-CF vector [22]. This was linearized so that the targeting fragment was at one end and telomeric sequences at the other. Plasmid DNA within the vector is marked in red. Site-specific integration (crossed lines) results in the deletion of approximately 100 kb of DNA between the target sequence and the telomere (Additional data file 2). Telomeric sequences supplied by the vector are shown as horizontal arrowheads. Expression of neor is under control of the rDNA promoter (flagged) [file gb-2007-8-3-r37-S1.pdf]
