# Supplementary material for: Repetitive DNA is associated with centromeric domains in Trypanosoma brucei but not Trypanosoma cruzi
Source: Genome Biol. 2007 Mar 12;8(3):R37. doi: 10.1186/gb-2007-8-3-r37 (PMC1868937; doi:10.1186/gb-2007-8-3-r37)

Additional Data File 2.

Generating a truncated version of the small homologue of *T. cruzi* chromosome 1.

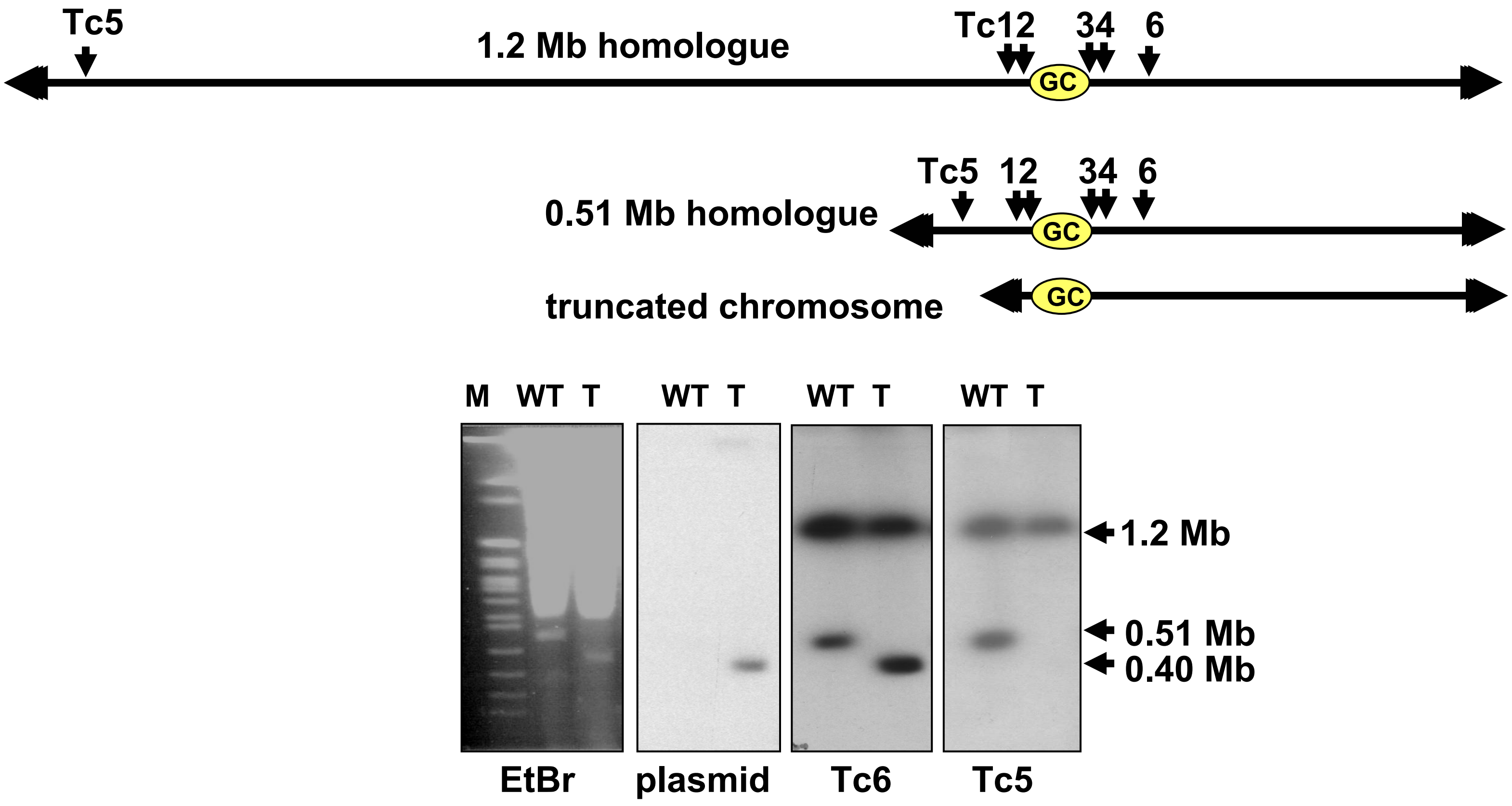

Supplement: Additional data file 2 — The 11 kb GC-rich strand-switch domains are shown (yellow oval). Arrows (Tc1-Tc4) indicate positions of the target sequences used for fragmentation (Additional data file 5). Parasites were transfected with linearized vector DNA and transformants selected with G418 [22] (see Materials and methods). Chromosomal DNA from wild-type (WT) and transfected (T) parasites was separated by CHEFE with S. cerevisiae markers (M) and analyzed by Southern blotting using plasmid, Tc5 and Tc6 radiolabelled probes. Autoradiographs are shown, together with an ethidium bromide (EtBr) stained gel, overexposed to show the truncated chromosome. In the example shown, integration at the Tc1 locus of the 0.51 Mb chromosome, in the direction of polycistronic transcription, results in deletion of approximately 100 kb of DNA between the target sequence and the telomere. Hybridization identifies the larger (1.2 Mb) and smaller homologues (0.51 Mb) and the truncated product (0.40 Mb). Plasmid DNA is incorporated into truncated chromosomes as part of the integration process (Additional data file 1) [file gb-2007-8-3-r37-S2.pdf]
