# Supplementary material for: Repetitive DNA is associated with centromeric domains in Trypanosoma brucei but not Trypanosoma cruzi
Source: Genome Biol. 2007 Mar 12;8(3):R37. doi: 10.1186/gb-2007-8-3-r37 (PMC1868937; doi:10.1186/gb-2007-8-3-r37)

Additional Data File 3.

Chromosome fragmentation demonstrates that the major size difference between the *T. cruzi* chromosome 1 homologues is the result of the insertion/deletion of 0.7 Mb of DNA into/from the left arm of the chromosome.

(A)

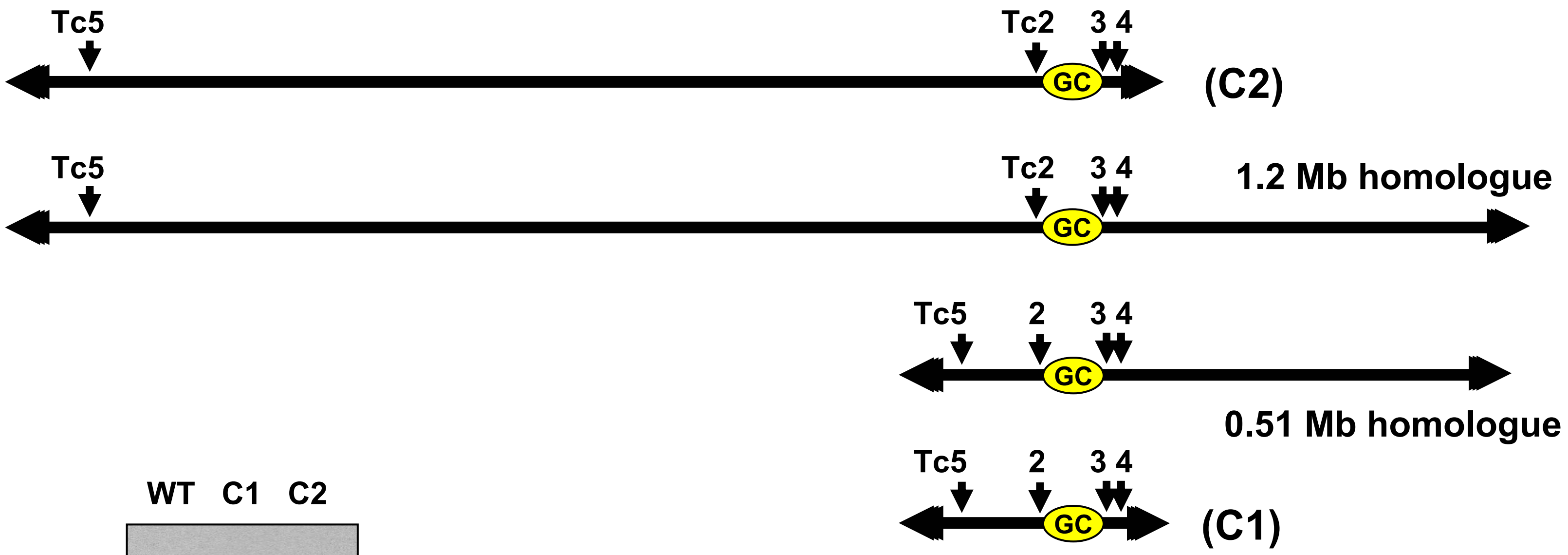

(B)

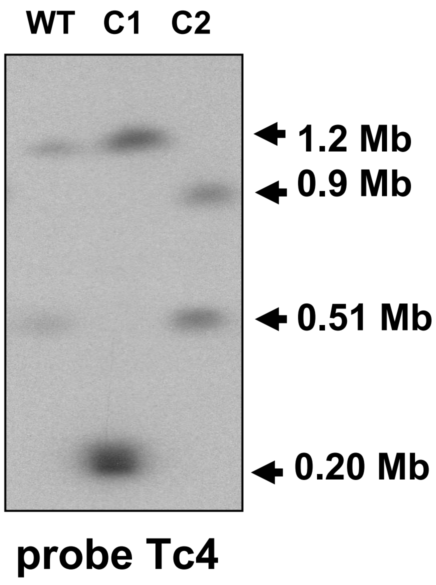

Supplement: Additional data file 3 — (a) Schematic showing the 0.51 Mb and 1.2 Mb homologues and their respective truncated products C1 (0.2 Mb) and C2 (0.9 Mb), with the locations of probes Tc2-Tc5 (Additional data file 5). (b) Autoradiograph illustrating the deletion of the right arms of each chromosome homologue following integration of the fragmentation vector at ORF Tc4. Two clones were isolated after transfection and chromosomal DNA from wild-type (WT) and both clones (C1 and C2) were separated by CHEFE and analyzed by Southern blotting using radiolabeled probe Tc4. Hybridization identifies the larger homologue (1.2 Mb), the smaller homologue (0.51 Mb) and their respective truncated products (0.9 Mb and 0.2 Mb). We had previously shown that probe Tc5 is located a similar distance (approximately 50 kb) from the end of both homologues [22] [file gb-2007-8-3-r37-S3.pdf]
