# Supplementary material for: Repetitive DNA is associated with centromeric domains in Trypanosoma brucei but not Trypanosoma cruzi
Source: Genome Biol. 2007 Mar 12;8(3):R37. doi: 10.1186/gb-2007-8-3-r37 (PMC1868937; doi:10.1186/gb-2007-8-3-r37)
Supplement: Additional data file 4 — T. brucei procyclic cultures were treated with 500 μM etoposide and chromosomal DNA isolated and fractionated using a Bio-Rad CHEF Mapper system. Membranes were hybridized with probes Tb1-Tb17, the GeneDB systemic names of which are shown in Additional data file 5. The positions of probes (arrows) and locations of the putative centromeric regions (yellow ovals) are indicated. Red arrows indicate INGI/DIRE retroelements and green arrows identify putative ORFs and the implied direction of transcription. The locations of the AT-rich repeat arrays are highlighted (striped box) and the %GC contents across the regions are shown. GeneDB systemic names of ORFs adjacent to the arrays are given and allow the repeat arrays to be mapped onto the chromosomal contigs. Lane N, non-treated parasites; lane E, etoposide-treated. Chromosome sizes were calculated using S. cerevisiae (0.2-2.2 Mb) and Hansenula wingei (1.0-3.1 Mb) chromosome markers (Bio-Rad). In most cases, sequence contigs do not extend to the ends of chromosomes. Gaps in the sequence of AT-rich arrays in chromosomes 3, 6 and 8 are indicated by asterixes and double slashes. We have also found, using long-range restriction mapping (Figures 4b and 5bB), that the 'centromeric-domains' of chromosomes 1 and 4 are larger than predicted by the genome sequence [file gb-2007-8-3-r37-S4.pdf]

Additional Data File 4.

Mapping of etoposide-mediated Topo-II cleavage sites in *T. brucei* chromosomes 1-8.

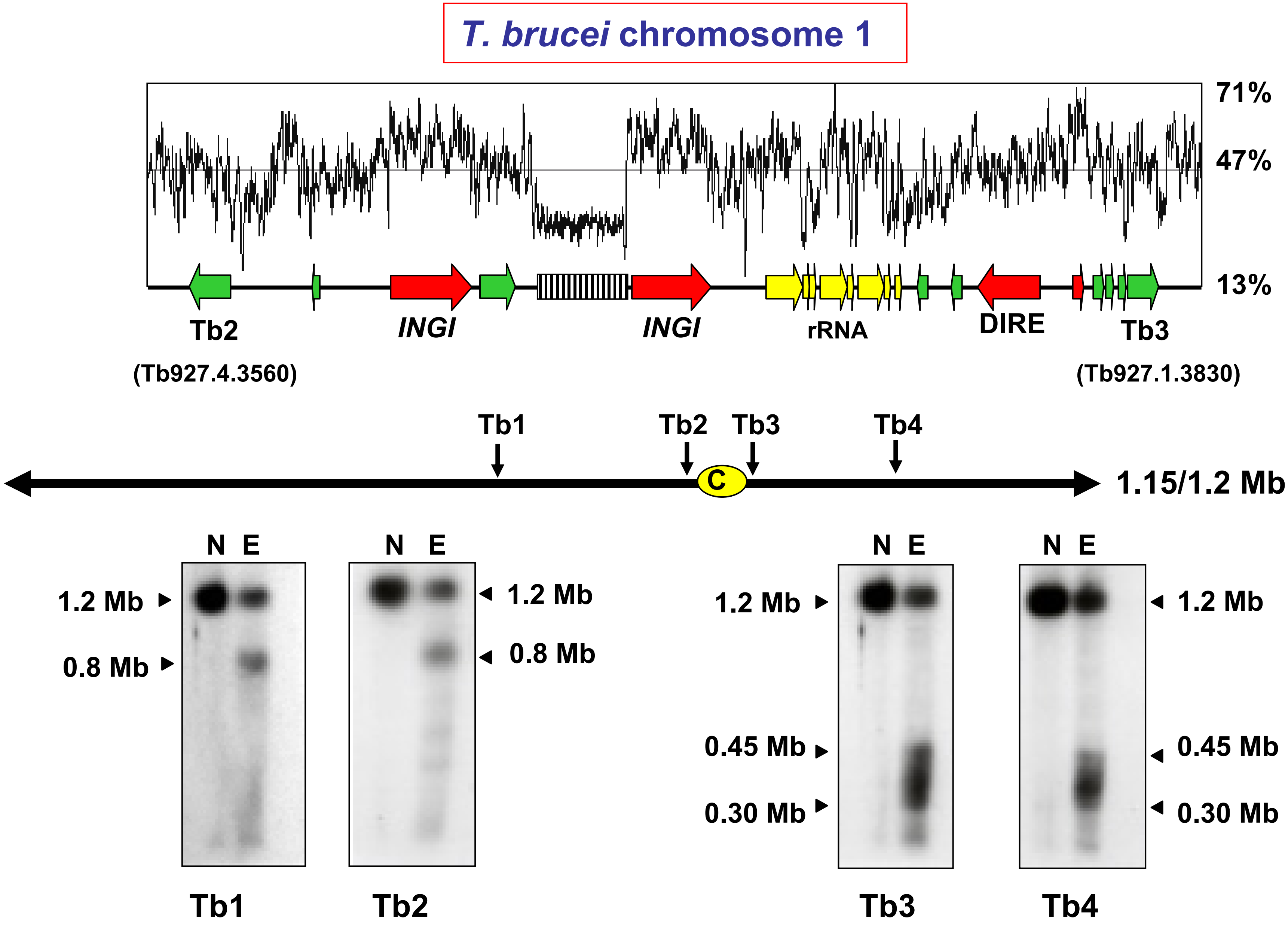

T. brucei chromosome 2

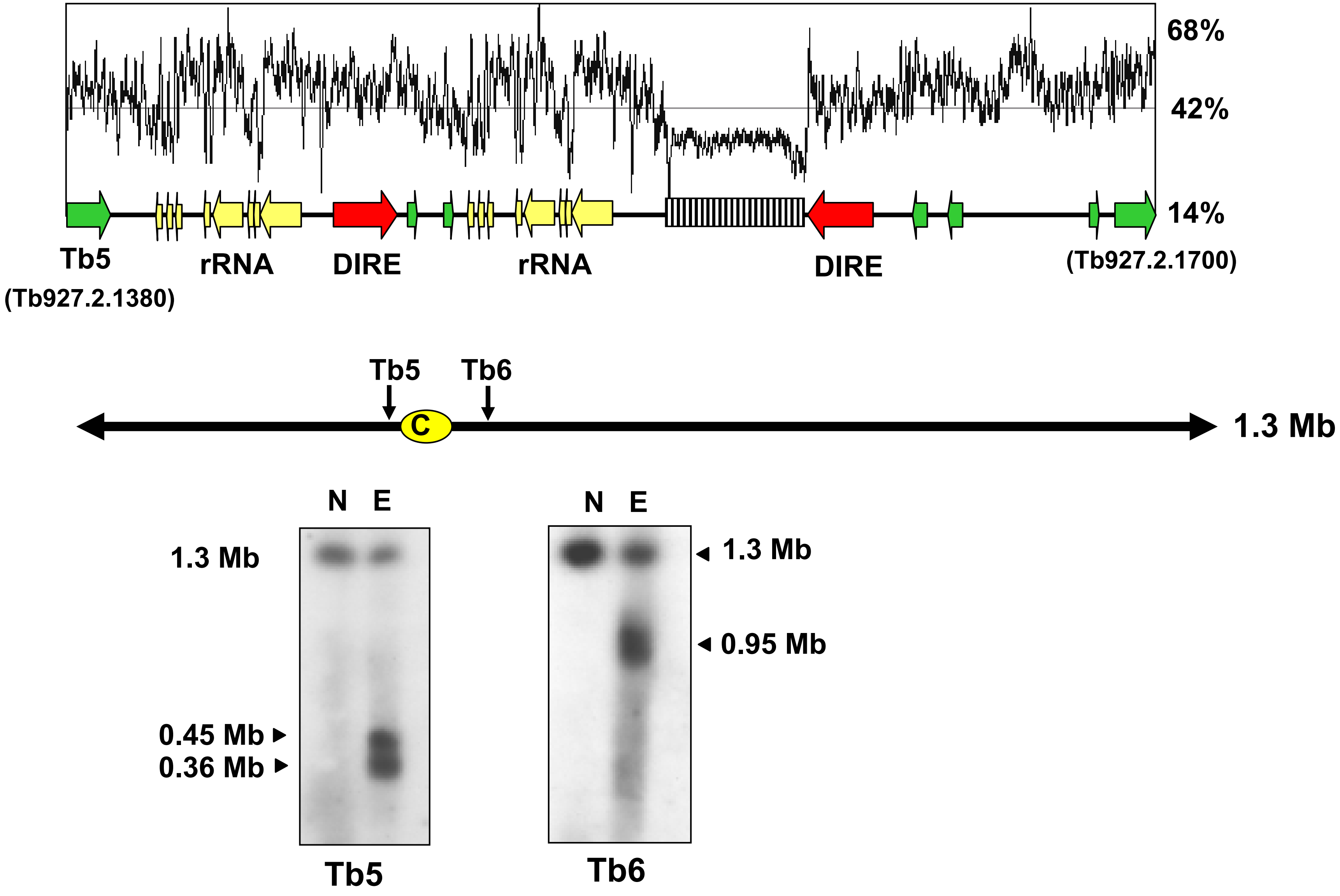

T. brucei chromosome 3

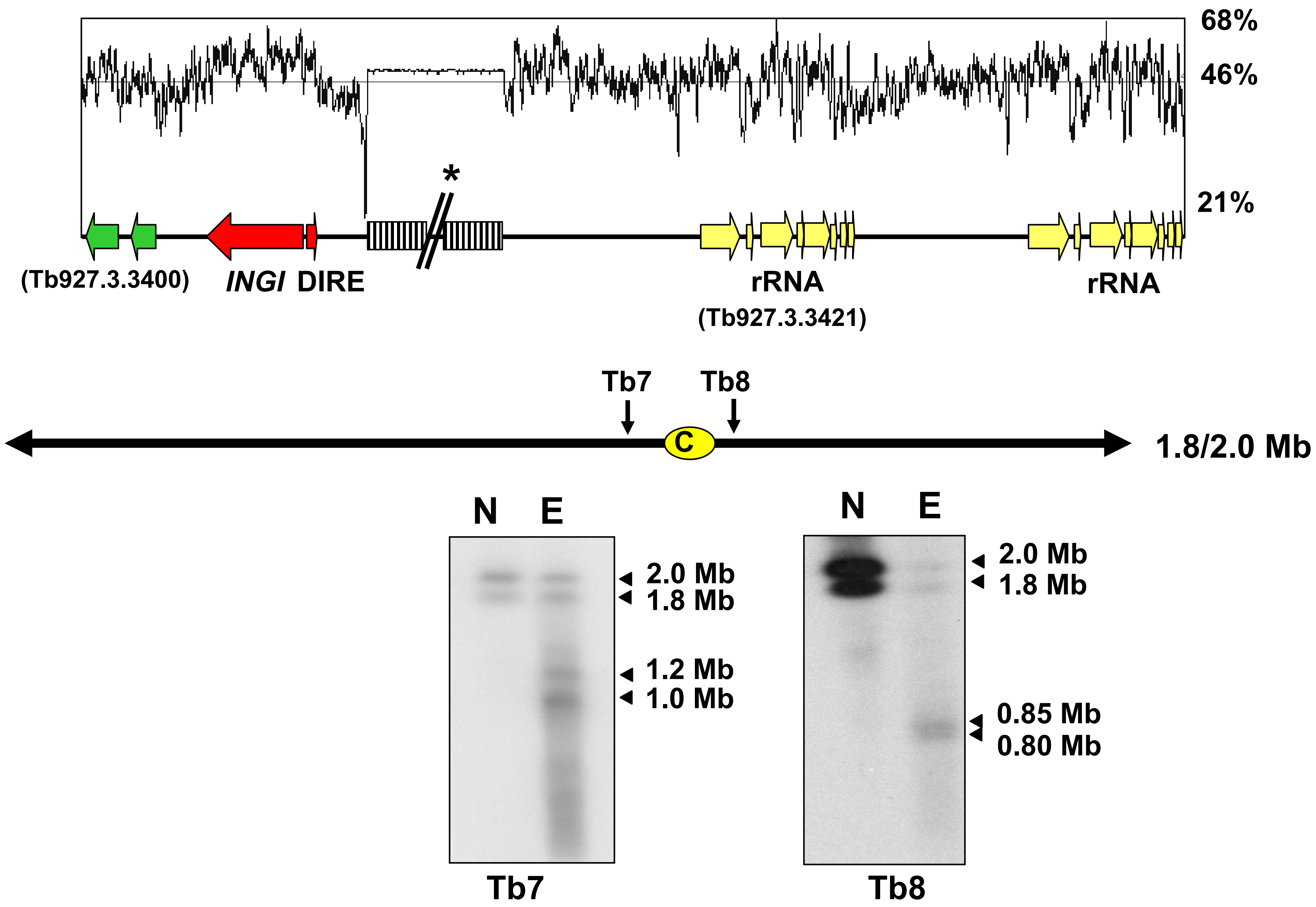

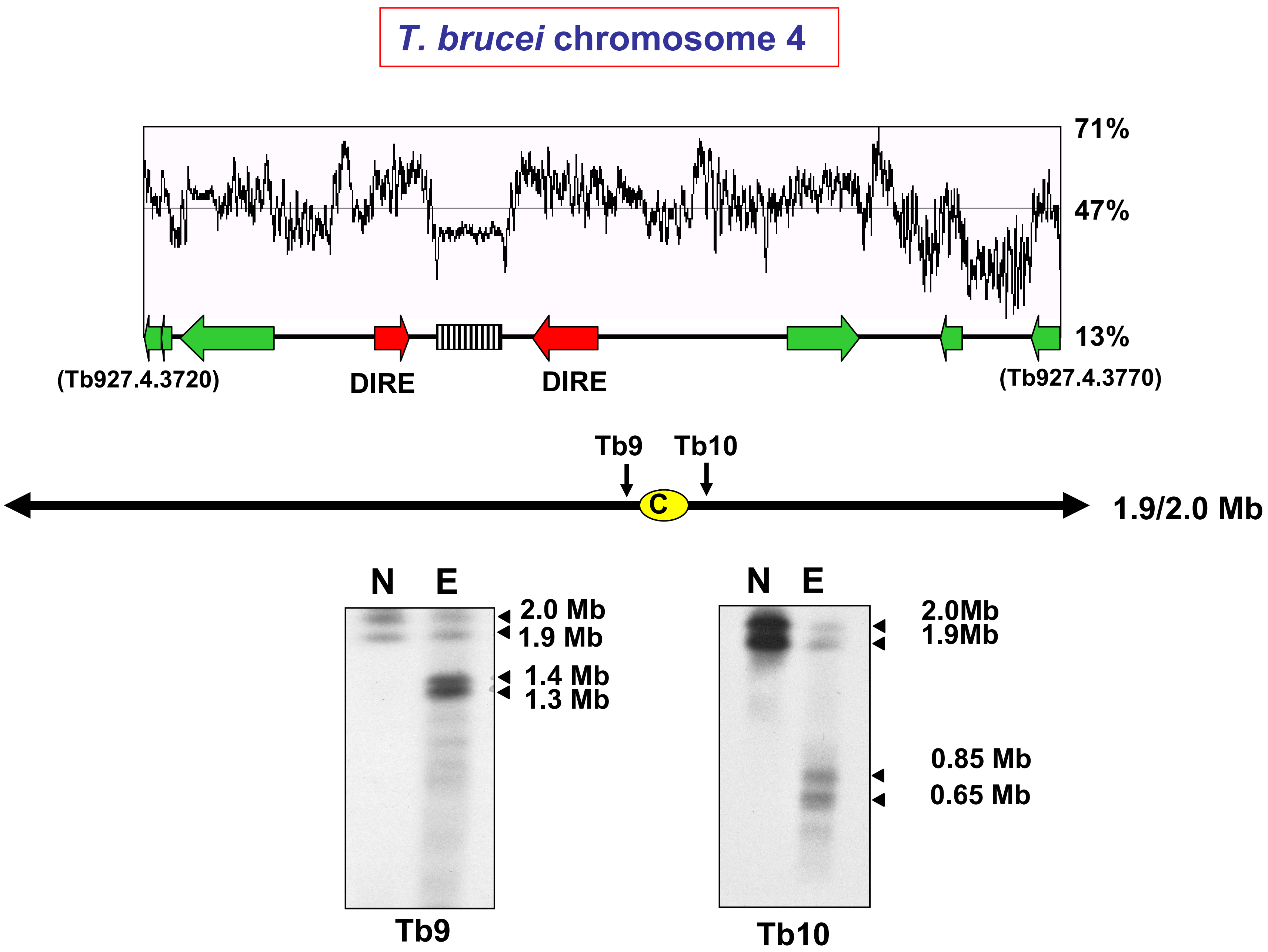
T. brucei chromosome 4
71%
47%
13%
(Tb927.4.3720)
DIRE
DIRE
(Tb927.4.3770)
Tb9
Tb10
C
1.9/2.0 Mb
N
E
2.0 Mb
1.9 Mb
1.4 Mb
1.3 Mb
Tb9
N
E
2.0Mb
1.9Mb
0.85 Mb
0.65 Mb
Tb10

T. brucei chromosome 5

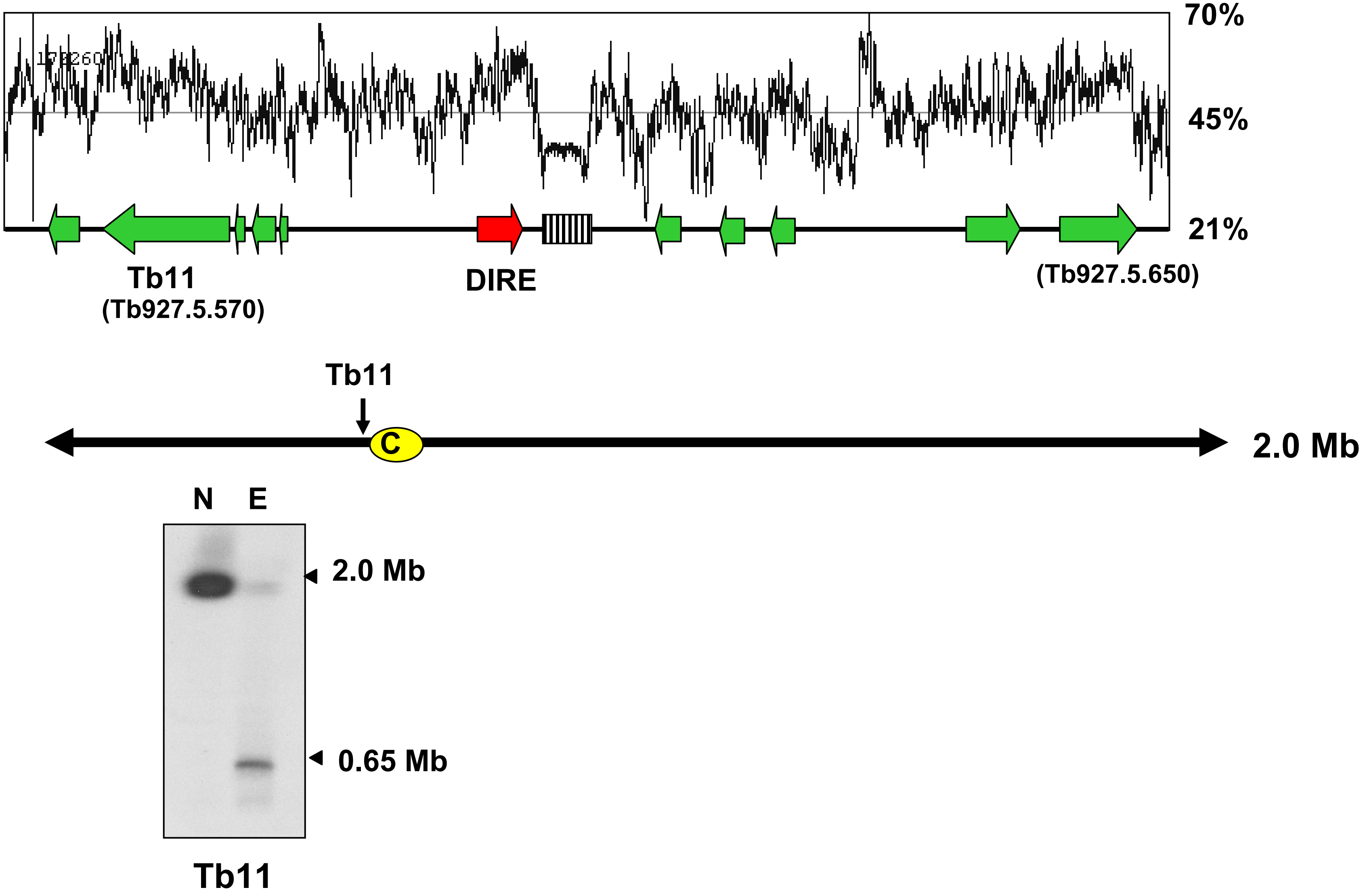

T. brucei chromosome 6

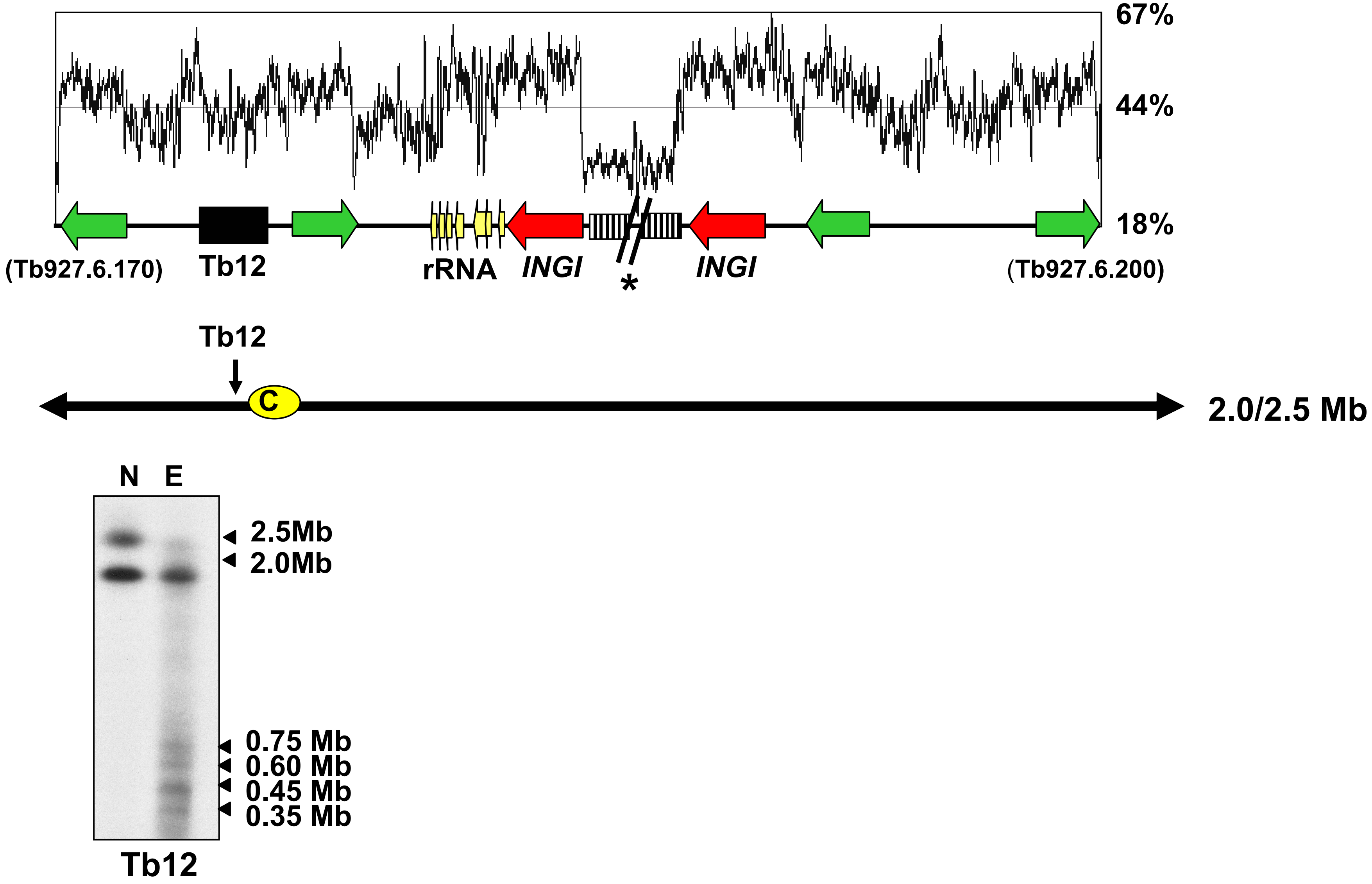

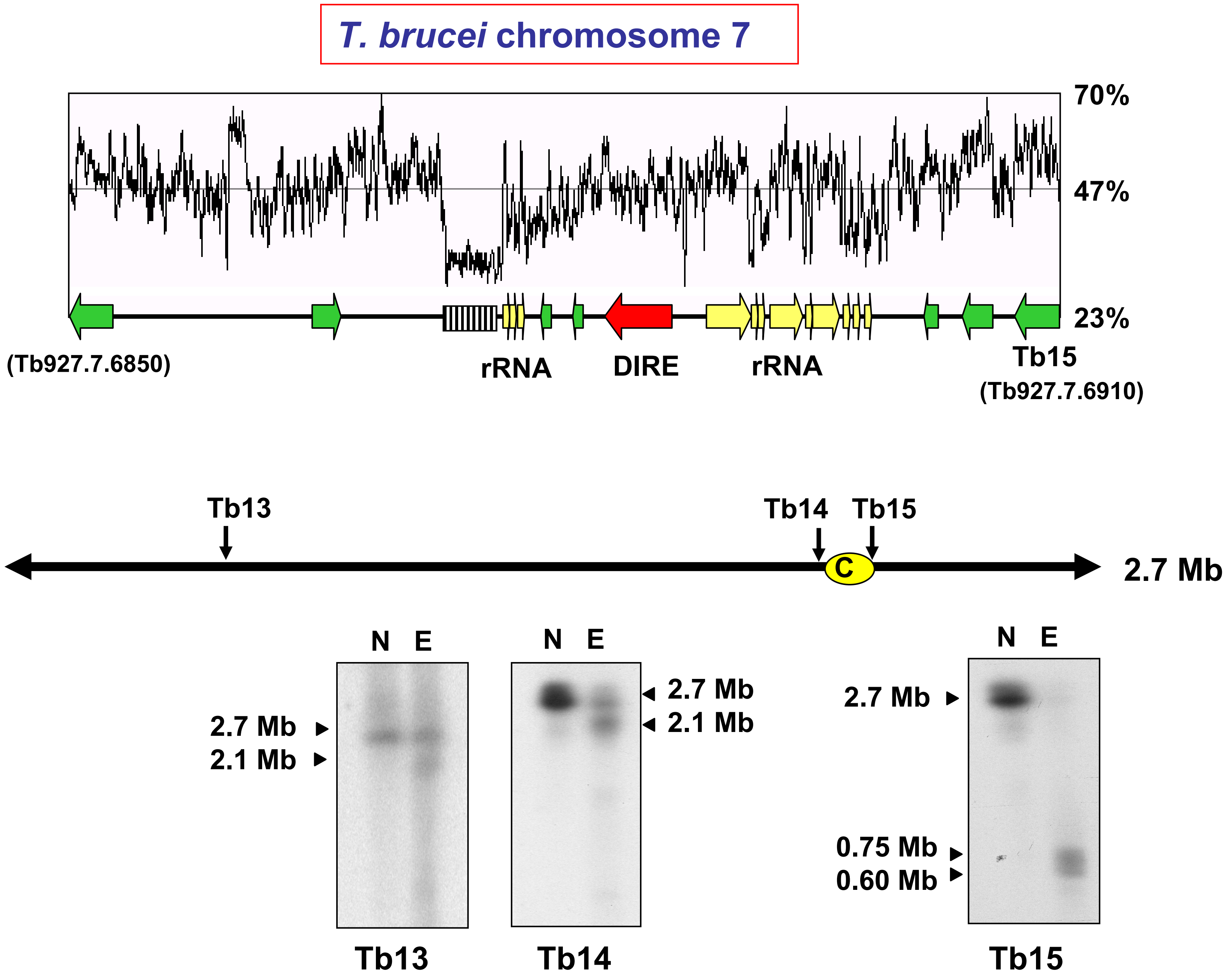
T. brucei chromosome 7
70%
47%
23%
(Tb927.7.6850)
rRNA
DIRE
rRNA
Tb15
(Tb927.7.6910)
Tb13
Tb14
Tb15
C
2.7 Mb
N
E
2.7 Mb
2.1 Mb
Tb13
N
E
2.7 Mb
2.1 Mb
Tb14
N
E
2.7 Mb
0.75 Mb
0.60 Mb
Tb15

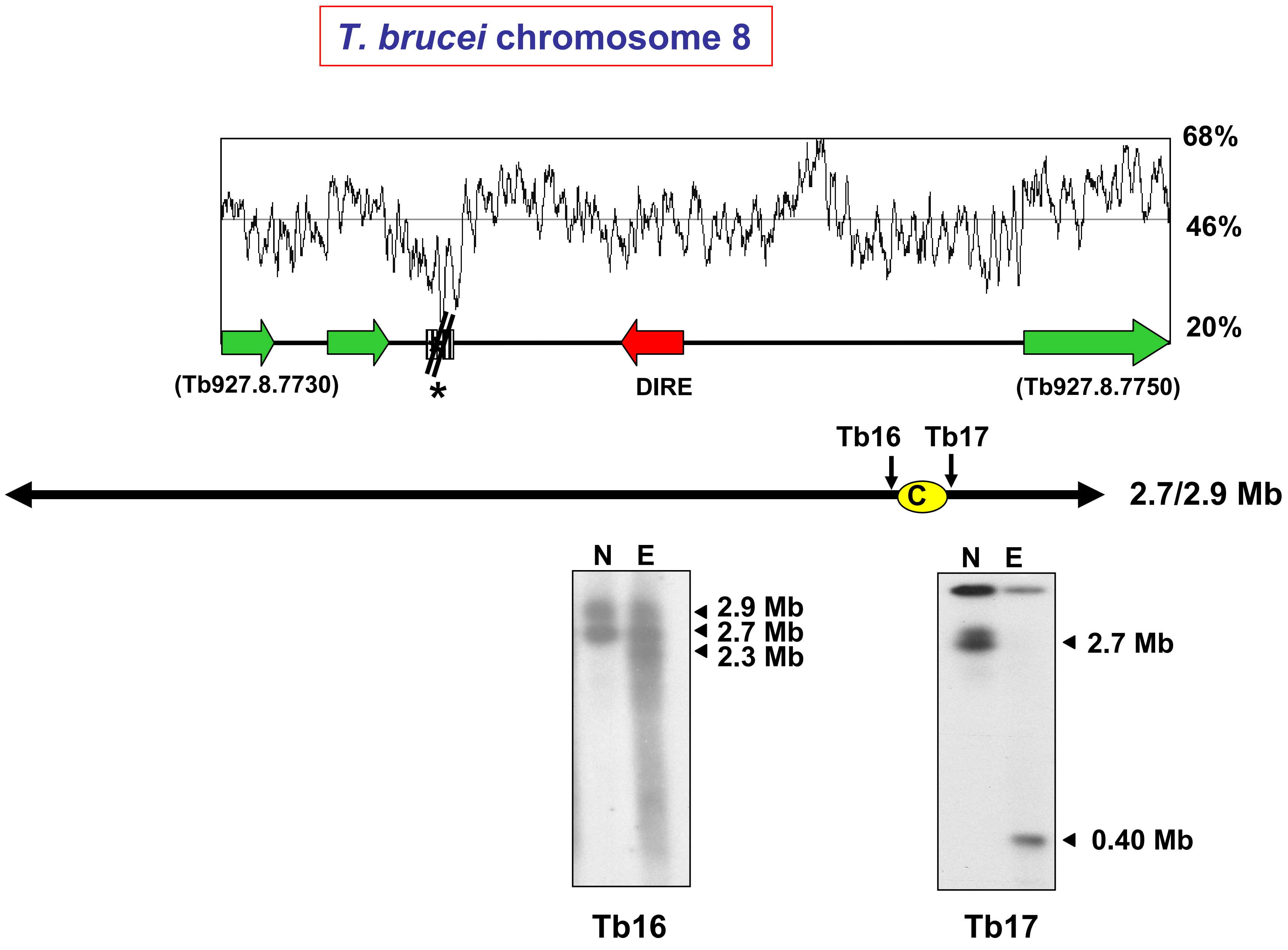

T. brucei chromosome 8
68%
46%
20%
(Tb927.8.7730)
*
DIRE
(Tb927.8.7750)
Tb16
Tb17
C
2.7/2.9 Mb
N
E
2.9 Mb
2.7 Mb
2.3 Mb
N
E
2.7 Mb
0.40 Mb
Tb16
Tb17
