# Supplementary material for: Repetitive DNA is associated with centromeric domains in Trypanosoma brucei but not Trypanosoma cruzi
Source: Genome Biol. 2007 Mar 12;8(3):R37. doi: 10.1186/gb-2007-8-3-r37 (PMC1868937; doi:10.1186/gb-2007-8-3-r37)
Supplement: Additional data file 5 — T. cruzi (Tc1-12) and T. brucei (Tb1-18) probes used in this study, together with their GeneDB systemic names [file gb-2007-8-3-r37-S5.pdf]

	Chromosome	GeneDB systemic name
Tc1	1	Tc00.1047053508707.160
Tc2	1	Tc00.1047053508707.190
Tc3	1	Tc00.1047053508707.320
Tc4	1	Tc00.1047053511021.30
Tc5	1	Tc00.1047053511019.99
Tc6	1	Tc00.1047053511021.80
Tc7	3	Tc00.1047053506529.660
Tc8	3	Tc00.1047053506529.600
Tc9	3	Tc00.1047053506529.508
Tc10	3	Tc00.1047053506529.340
Tc11	3	Tc00.1047053398147
Tc12	3	Intergenic sequence*

*see Materials and Methods

***T. brucei* probes**	**Chromosome**	**GeneDB systemic name**
Tb1	1	Tb927.1.2340
Tb2	1	Tb927.1.3560
Tb3	1	Tb927.1.3830
Tb4	1	Tb927.1.4720
Tb5	2	Tb927.2.1380
Tb6	2	Tb927.2.2090
Tb7	3	Tb927.3.3300
Tb8	3	Tb927.3.3550
Tb9	4	Tb927.4.3690
Tb10	4	Tb927.4.3820
Tb11	5	Tb927.5.570
Tb12	6	Intergenic sequence (35563-36918)*
Tb13	7	Tb927.7.1690
Tb14	7	Tb927.7.6820
Tb15	7	Tb927.7.6910
Tb16	8	Tb927.8.7710
Tb17	8	Tb927.8.7770
Tb18	4	Tb927.4.1480

* see Materials and Methods

Additional Data File 5.
List of *T. cruzi* (Tc1-12) and *T. brucei* (Tb1-18) probes used in this study, together with their GeneDB systemic names.
